# A comparison of the clinical effectiveness and cost of specialised individually delivered parent training for preschool attention-deficit/hyperactivity disorder and a generic, group-based programme: a multi-centre, randomised controlled trial of the New Forest Parenting Programme versus Incredible Years

**DOI:** 10.1007/s00787-017-1054-3

**Published:** 2017-10-30

**Authors:** Edmund J. S. Sonuga-Barke, Joanne Barton, David Daley, Judy Hutchings, Tom Maishman, James Raftery, Louise Stanton, Cathy Laver-Bradbury, Maria Chorozoglou, David Coghill, Louisa Little, Martin Ruddock, Mike Radford, Guiqing Lily Yao, Louise Lee, Lisa Gould, Lisa Shipway, Pavlina Markomichali, James McGuirk, Michelle Lowe, Elvira Perez, Joanna Lockwood, Margaret J. J. Thompson

**Affiliations:** 1 Academic Unit of Psychology, University of Southampton, Southampton, SO17 IBJ UK; 2 Department of Experimental Clinical and Health Psychology, Ghent University, Ghent, Belgium; 3 North Staffordshire Combined Healthcare NHS Trust, Stoke-on-Trent, UK; 4 Division of Psychiatry and Applied Psychology, University of Nottingham, Nottingham, NG7 2TR UK; 5 NIHR MindTech, Institute of Mental Health, University of Nottingham, Nottingham, NG7 2TR UK; 6 Centre for Evidence Based Early Intervention, School of Psychology, Bangor University, Bangor, UK; 7 Southampton Clinical Trials Unit, University of Southampton, Southampton, UK; 8 Primary Care and Population Sciences, University of Southampton, Southampton, UK; 9 CAMHS, Solent NHS Trust, Better Care Centre, Southampton, UK; 10 Southampton Health Technology Assessment Centre (SHTAC), Faculty of Medicine, University of Southampton, Southampton, UK; 11 Departments of Paediatrics and Psychiatry, Faculty of Medicine, Dentistry and Health Sciences, University of Melbourne, Melbourne, Australia; 12 Division of Neuroscience, School of Medicine, University of Dundee, Dundee, UK; 13 Department of Child and Adolescent Psychiatry, Institute of Psychiatry, Psychology and Neuroscience, King’s College London, 16 De Crespigny Park, Camberwell, London, SE5 8AF UK

**Keywords:** ADHD, New Forest, Parenting Programme, NFPP, Parenting, Incredible Years, IY

## Abstract

The objective of this study is to compare the efficacy and cost of specialised individually delivered parent training (PT) for preschool children with attention-deficit/hyperactivity disorder (ADHD) against generic group-based PT and treatment as usual (TAU). This is a multi-centre three-arm, parallel group randomised controlled trial conducted in National Health Service Trusts. The participants included in this study were preschool children (33–54 months) fulfilling ADHD research diagnostic criteria. New Forest Parenting Programme (NFPP)—12-week individual, home-delivered ADHD PT programme; Incredible Years (IY)—12-week group-based, PT programme initially designed for children with behaviour problems were the interventions. Primary outcome—Parent ratings of child’s ADHD symptoms (Swanson, Nolan & Pelham Questionnaire—SNAP-IV). Secondary outcomes—teacher ratings (SNAP-IV) and direct observations of ADHD symptoms and parent/teacher ratings of conduct problems. NFPP, IY and TAU outcomes were measured at baseline (T1) and post treatment (T2). NFPP and IY outcomes only were measured 6 months post treatment (T3). Researchers, but not therapists or parents, were blind to treatment allocation. Analysis employed mixed effect regression models (multiple imputations). Intervention and other costs were estimated using standardized approaches. NFPP and IY did not differ on parent-rated SNAP-IV, ADHD combined symptoms [mean difference − 0.009 95% CI (− 0.191, 0.173), *p* = 0.921] or any other measure. Small, non-significant, benefits of NFPP over TAU were seen for parent-rated SNAP-IV, ADHD combined symptoms [− 0.189 95% CI (− 0.380, 0.003), *p* = 0.053]. NFPP significantly reduced parent-rated conduct problems compared to TAU across scales (*p* values < 0.05). No significant benefits of IY over TAU were seen for parent-rated SNAP, ADHD symptoms [− 0.16 95% CI (− 0.37, 0.04), *p* = 0.121] or parent-rated conduct problems (*p* > 0.05). The cost per family of providing NFPP in the trial was significantly lower than IY (£1591 versus £2103). Although, there were no differences between NFPP and IY with regards clinical effectiveness, individually delivered NFPP cost less. However, this difference may be reduced when implemented in routine clinical practice. Clinical decisions should take into account parental preferences between delivery approaches.

## Introduction

Preschool attention-deficit/hyperactivity disorder (ADHD) impacts daily functioning [1] and predicts future burden [2]. Parent training (PT), which aims to teach parents ways to improve their children’s behaviour using social learning theory-based principles and techniques [3, 4], is the first-line treatment for preschool ADHD [5]. Some programmes are delivered individually, on a one-to-one basis [3]; others are delivered to small groups of parents [7, 8]. The National Institute for Health and Care Excellence (NICE) currently recommends group-based PT for ADHD in young children [5] based on an assumption that it is no less efficacious and likely to be cheaper than individually delivered PT. At the time that this recommendation was published, there was insufficient evidence from trials evaluating group- and individual-PT approaches in the treatment of ADHD and so NICE extrapolated from evidence from studies of PT for conduct problems, when giving this advice [5]. Given that ADHD and conduct problems, although often co-existing, are different disorders with a different aetiology, pathogenesis and prognosis, which require different treatments, establishing the relative efficacy and cost-effectiveness of individual and group approaches for preschool ADHD is an important mental health research priority.

To achieve this, here we present the Comparison of Preschool Parenting Interventions (COPPI) trial, the first randomised controlled trial (RCT) directly comparing the efficacy and cost-effectiveness of a PT programme delivered on a one-to-one basis—the New Forest Parenting Programme (NFPP) [6] and a group-based PT intervention of the sort recommended by NICE—Incredible Years (IY) [9] for the treatment of ADHD.

NFPP was selected for the trial, as it is the only widely available individually delivered programme developed as a PT intervention specifically for preschool children with ADHD. It is delivered at home on a one-to-one basis and tailored to the parent. It has four main therapeutic elements: (i) Psycho-education about ADHD; (ii) ADHD-tailored strategies to promote proactive parenting/better communication; learning to wait, and cueing the child into a change of task; (iii) play-based enhancement of the parent–child relations and; (iv) attention training through structured games and teachable moments [10–12]. RCTs support the value of NFPP with regard to reducing ADHD and conduct problems [6, 11, 12] as well as improving parental mental health and parent–child interaction, at least in the short term [12].

During the design of the study when considering which group-based PT comparator to use, we initially considered adapting the NFPP for use with groups to allow a direct comparison of group and one-to-one approaches of the same PT approach. However, as this would require a whole programme of research and development, before the current trial could be initiated, we decided on a more pragmatic approach—to contrast NFPP with a group-based approach that already had an established evidence base and was recommended by NICE [5]. We chose IY for this role because it is widely used in the UK and is an example used by NICE to illustrate the sort of programme it recommended for children with preschool ADHD. IY comprises a series of developmentally based interventions for parents, children and teachers, derived from reinforcement and cognitive social learning principles. In the current trial, we used the 12-session IY Toddler programme [13]. This combined problem-solving, videotape modeling, role play practices, support network building and on-going home assignments to facilitate: (i) child-directed play to promote positive relationship between parent and child; (ii) social, emotional and persistence coaching to promote language and attention focus; (iii) praise and incentives to promote appropriate child behaviours; (iv) predictable routines and effective limit setting; and (v) proactive strategies to manage misbehaviour. A large body of literature supports its value with regard to conduct problems and the IY-basic parent programme has also shown promise in treating ADHD behaviours [14–17].

Our research questions were: Is NFPP superior to IY and treatment as usual (TAU) in terms of reduction of parent-rated ADHD symptoms (our primary outcome)? Do any observed effects generalise to teacher-rated and directly observed ADHD symptoms and extend to parent/teacher-rated conduct problems (secondary outcomes)? What are the costs and cost-effectiveness of each type of treatment?

## Methods

### Design

A multi-centre three-arm, parallel randomised controlled trial comparing NFPP with both IY and TAU, for preschool children with a research diagnosis of ADHD.

### Participants

Participants were enlisted (February 2012 to January 2014) at three UK sites: (i) University of Southampton [with Solent NHS Trust (Southampton and Portsmouth Cities)]; (ii) North Staffordshire Combined Health Care NHS Trust; (iii) University of Nottingham (with Nottingham City Care/Nottinghamshire County Health Partnerships). Ethical approval from the NHS Research Ethics Committee and site-specific approvals from the contributing sites were received. After being given a study description, parents provided informed consent. Participants were recruited and randomised in five (Nottingham and North Staffs) or six (Southampton) tranches in a way that allowed one or more IY groups to run at each site. Our aim was for our sample to be as representative of the entire preschool ADHD population as possible and therefore children, including those with co-occurring problems or living in difficult circumstances, were recruited from a wide range of sources. Sources included health visitors, Sure Start professionals, speech therapists, paediatric and child psychiatry clinics and adult mental health services. Posters, radio advertisements and social media were also employed. Children were included if: (i) they were between 2 years 9 months and 4 years 6 months old; (ii) had a parent/caregiver aged 18 years or over; (iii) screened positive for ADHD symptoms (score≥ 20) on the Werry-Weiss-Peters Activity Rating Scale (WWP) [18] and; (iv) were given an ADHD research diagnosis of any sub-type based on the parent DISC-IV-ADHD Scale [19]. To further ensure the inclusion of a wide-ranging representative sample of ADHD preschool children, cases were only excluded if they had: (i) a full clinical diagnosis of autism spectrum disorder; (ii) were severely delayed developmentally (18 months or more behind their chronological age on the Parent Involvement Project (PIP) Developmental Scales [20]; (iii) had a main caregiver with a serious mental illness (e.g., psychosis). They were also excluded for practical reasons including: (iv) if children were in short- to medium-term foster care placements; (v) on the Child Protection Register or (vi) when their main carer had insufficient English language. Information concerning exclusion was available to referrers and reassessed at the screening and first assessment visit.

### Allocation and blinding

After all baseline (T1) measures were completed, participants were block randomised into study arms by the Southampton Clinical Trials Unit using the TENALEA [see www.tenalea.com] system [3 (NFPP): 3 (IY): 1 (TAU) ratio] to ensure power for the comparison of the two treatment arms. Stratification was by site and tranche. Parents and therapists were not blinded to treatment allocation. However, to protect blinding for all other members of the team including statisticians and researchers collecting and coding direct observations, only site PIs and designated administrative staff liaised with the trials unit and participants, with regard to allocation. Families were informed of the need to maintain blindness. This meant that researchers who collected outcome measures at T2 and T3 (see below) were, as far as possible, blind to treatment allocation. Teachers were also potentially blind to allocation. The coding of the observation data (which was videoed) was done by a researcher who had not met the family and was unaware of the group allocation. Inter-centre reliability of observation data was high.

### Interventions

The general principles and structure of NFPP and IY are described in the Introduction. More detailed procedural information is given here. Both interventions are described in the published protocol [21].

#### NFPP

Prior to the trial, we conducted a detailed analysis of the content of the NFPP programme. This led to its extension from an 8- to a 12-week version [22, 23], which meant it could be delivered at a slower pace with more emphasis on reinforcing messages to help parents with literacy or intellectual problems. New modules addressing: (a) child sleep problems, learning difficulties and language problems and (b) parental mental health problems and learning difficulties, were added and employed if needed. Two parent–child sessions were videoed to provide interactive feedback. Each session lasted approximately 1.5 h. Handouts, DVD/CDs and other resources were provided. Sessions were videoed for supervision purposes [12].

#### IY

This was delivered in venues as local to the families as possible, in clinics or *Sure Start* centres. In each study centre, weekly sessions of ~2–2.5 h duration were run for 12 weeks (with breaks for half-term school holidays). Handouts, CDs, books and gifts were distributed. Lunches and crèches (a facility where parents could leave their children while they attended training) were provided by child-care experts. Transport was also provided if needed (these factors are part of the standard IY protocol for a trial). Parents received weekly phone calls from therapists and, where possible, parents who missed a session received a home visit. This constituted 9% of IY sessions. Pairs of therapists worked together in each group to deliver the therapy. All sessions were videoed for supervision purposes.

### Training and supervision

All therapists appointed were naïve at the beginning to the programme to which they were allocated. The backgrounds of the therapists varied (nurses, social workers, psychologists, family support workers). All had a background in working with children and parents. For both programmes, therapists received regular supervision following 21 h of initial training according to standard protocols. Between January and December 2012, NFPP supervision consisted of 1-h weekly phone calls with all the therapists on-line. For the remainder of the trial (Jan 2013 to March 2014), therapists at each site had one joint monthly phone call supplemented by a 3-h monthly face-to-face session to review DVDs. All therapists also met twice as a group for 5 h with MJJT and CLB. IY supervision was delivered face-to-face by one of the four mentors approved by the programme developer. Each therapist should have received 32, 2-h supervision sessions covering a trial group exercise (four sessions) and regular sessions across tranches (12 in tranche 1, four in tranches 2, 3 and 4 and two in tranches 5 and 6). There were also four, 4-h meetings for all therapists. However, therapists occasionally missed supervisions and new therapists who joined the project at all three sites received supplementary sessions in addition to initial training and on-going planned supervision. However, due to staff changes this did not equate to the planned schedule and new staff did not have an opportunity to trial the programme before participating in research groups.

### Treatment as usual

Children in TAU received the standard patterns of preschool ADHD care available in their region. In two of the regions, there was little provision for preschool ADHD while in one region provision might include parenting education and training.

### Treatment fidelity

The proportion of therapeutic content delivered for each intervention was measured using therapist-completed checklists tailored to programme-specific content. For NFPP and IY, respectively, these were assessed by MJJT and JH. IY checklists were completed for two sites only as the therapists in the third site did not send completed checklists to be assessed despite repeated encouragement. Video-tapes of individual sessions were also watched to allow supervisors to rate fidelity of therapists to the programme content and also to use in supervision. This was completed independently of blinded coding of video-tapes of the child’s behaviour and parent–child interaction that were used as outcome variables at T1, T2 and T3.

### Measures

#### Assessment schedule

Trained researchers collected data at three time points: Baseline (T1), post treatment (T2 approximately week 14) and at six-month follow-up after treatment (T3). The diagnostic screen was completed at home, in a clinic or by telephone. Baseline measures were taken at the family home at T1 prior to randomisation. For ethical reasons, T2 was the last follow-up for TAU participants who were subsequently offered a community-based PT programme. This was because the ethical committee judged that a potentially effective therapy should not be withheld from this group for longer than absolutely necessary.

### Screen and diagnostic interviews

#### Eligibility assessments

(i) Werry-Weiss-Peters Questionnaire [18] is a 27-item parent completed questionnaire. The cut-off score of 20 identifies around 15–18% of the population [25] Cronbach’s alpha for this measures in this sample was 0.87. (ii) Diagnostic Interview Schedule for Children—Version IV DISC-IV-ADHD Scale [19] is a well-validated structured interview used to diagnose ADHD according to the Diagnostic and Statistical Manual of Mental Health Disorders (DSM-IV) criteria using parental reports of symptoms in home and school settings; (iii) Parent Involvement Project Developmental Charts **(**PIP) Developmental Scales [20] is a UK norm-based developmental checklist which identifies delay against milestones covering physical and social development, hand–eye coordination, play and language which was delivered in an interview format. Language delay and Developmental delay were deemed present when an individual was at least 6 months behind their chronological age in relation to at least one milestone.

### Outcome measures

*Swanson Nolan and Pelham* (SNAP)-IV–Parent (primary outcome), Teacher Scales (SNAP-IV-T: SNAP-IV-P) [26] are well-validated 26-item questionnaires measuring the full 18 DSM-5 ADHD symptoms (9 inattentive, 9 hyperactive/impulsive) as well as eight oppositional defiant disorder (ODD) symptoms. Items are rated for frequency on a four-point scale (0 = not at all, to 3 = very much). Cronbach’s alpha for these measures in this sample were parent ADHD = 0.89, parent ODD = 0.89, teacher ADHD = 0.96 and teacher ODD = 0.93.

Eyberg Child Behaviour Inventory (ECBI) is a well-validated parent-completed 36-item childhood problem behaviour inventory. Each item is rated on both a 7-point Intensity Scale (Never to Always) and a Yes–No Problem Scale. Children scoring 15 or more on the problem scale were deemed to have clinically significant problems [27]. Cronbach’s alpha for this measure in this sample was *Intensity* = 0.93 and Problem = 0.87.

Directly Observed Attention (DOA) [6] is derived using direct observation of 5-min episodes of child solo play on the ‘Little People Animal Sounds Zoo’ (which includes different activity zones). An index of attending to, and switching from, one zone to another was calculated (time on task/total number of switches from zone to zone). The measure has good psychometric properties [6]. In the current study, the task was videoed and an observer rated the behaviours against established codes. Inter-rater reliability between coders was high both within (0.85–0.96), and between centres (0.76–0.96).

Client Service Receipt Inventory (CSRI) [28] is a tool to retrospectively collect health economic data from parents. In addition, socio-demographic information, service-related and non-service-related cost data were collected including: care service use (health clinics, health visitors, GPs, paediatric and mental health services); extra educational provision (school nurses, educational psychologist); social services and parental time off work. Data were collected over a three-month window or ‘since the last CSRI’ (if measures were at T2 and T3). The CSRI has been used in a number of evaluations of child mental health care [28].

General Health Questionnaire (GHQ) [29] is a screener for common mood-related conditions such as depression and anxiety. Parents completed the 12-item versions with items scored from 0 to 3. Those with a score of 11 or more are deemed to have probable mental health problems. Cronbach’s alpha for this measure in this sample was 0.89.

### Sample size determination

The trial was primarily powered to answer two questions. Is NFPP superior to (i) IY, and (ii) TAU in terms of reductions in parent-rated ADHD symptoms? Previous trials supported a conservative estimate of 0.4 standard deviations between NFPP and IY based on the effect size of the NFPP in previous trials 0.87 [6] and 1.96 [12] and the results of a recent IY trial [7] and 0.5 SD between NFPP and TAU. This equated to a 0.28 (SD = 0.7) and 0.35 (SD = 0.7) change on the mean SNAP-IV-P ADHD score (primary outcome; 5% two-tailed test of significance and 80% power). An intra-class correlation of 0.08 between scores for parents treated in the same IY groups and a drop-out rate of 10% was assumed for both estimates (as this had been the drop-out rate in previous community trials NFPP [12] and IY [7]. The trial needed to recruit 141 individuals into each of the active treatment arms and 47 in TAU (total *n* = 329). Each centre had a recruitment target of approximately 110 families.

### Funding

This was an independent study funded by the National Institute for Health Research (NIHR) under its Programme Grants for Applied Research scheme (RP-PG-0108-10061 to Solent NHS Trust who were the grant holders and hosted the trial).

### Statistical analyses

Statisticians at Southampton Clinical Trials Unit followed a pre-specified statistical analysis plan (available on request) using SAS version 9.4 and STATA version 12.1. Missing data were assessed by comparing baseline characteristics for participants by availability of primary endpoint information and was assumed to be either missing at random/missing completely at random. Missing data was handled by multiple imputation with STATA with the incorporation of REALCOM [30, 31] for therapist clustering (burn in length 500, number of iterations 2500 and 10 multiple imputations). Based on the ITT population, mixed effects regression models (using realcomImpute, mi estimate and xtmixed commands in STATA) tested the superiority of NFPP over IY and TAU in terms of primary (parent ADHD ratings) and secondary analyses (teacher ADHD ratings, teacher and parent ratings of ODD and Direct Observation of Attention) at T2 and T3 separately. T1 scores, treatment arm, centre and tranche were entered as fixed effects and participant and therapists as random effects. Results are presented in terms of estimated least square mean differences [with 95% 2-sided Confidence Intervals (CIs)]. Sensitivity analyses were conducted using the complete case analysis set, a per protocol sample (excluding participants who breached trial protocol), and those attending eight or more treatment sessions. Although not in the original analysis plan, post hoc comparisons of IY and TAU were also made using the same models. All p values reported are 2-sided. A significance level of < 0.05 was considered statistically significant. ITT analyses are presented using multiple-imputed data, unless otherwise stated. Standard mean differences (SMD) were calculated as ad hoc effect size analyses.

### Cost analysis

Although we planned to conduct a cost utility analysis comparing NFPP and IY, this was premised on finding statistically significant differences between TAU and both NFPP and IY on the primary outcomes. Since the results (Table 2) showed very small, non-significant differences in outcomes between IY and NFPP arms, the focus shifted to establishing the difference in costs between NFPP and IY. This matters because NICE recommendations assumed lower cost per family for group as opposed to individual therapy. Taking a combined societal/NHS perspective, the cost of interventions, to the health service and the family, was estimated. Information about resources required to provide the interventions was collected using a time collection form (TCF) to record the time therapists spent in delivering the intervention inclusive of preparation and travel time. The provision of manuals, handouts, training and supervision and any necessary fidelity procedures were also costed. Resource use was combined with relevant 2013 unit cost data to estimate the costs of providing interventions. Two adjustments were made to remove trial-specific cost elements that would not apply in normal practice: (i) travel time to supervision in both arms was omitted and, (ii) as crèche costs were unusually high in one of the centres they were reduced to an average. Both of these elements were unreasonably high for IY, perhaps specifically reflecting the way that they were provided in the trial. These adjustments, therefore, reduced costs more for IY than NFPP (detailed information available on request). Given the diversity of provision, we were not in a position to estimate the service costs for the families who received TAU.

## Results

### Sample

**Fig. 1:**
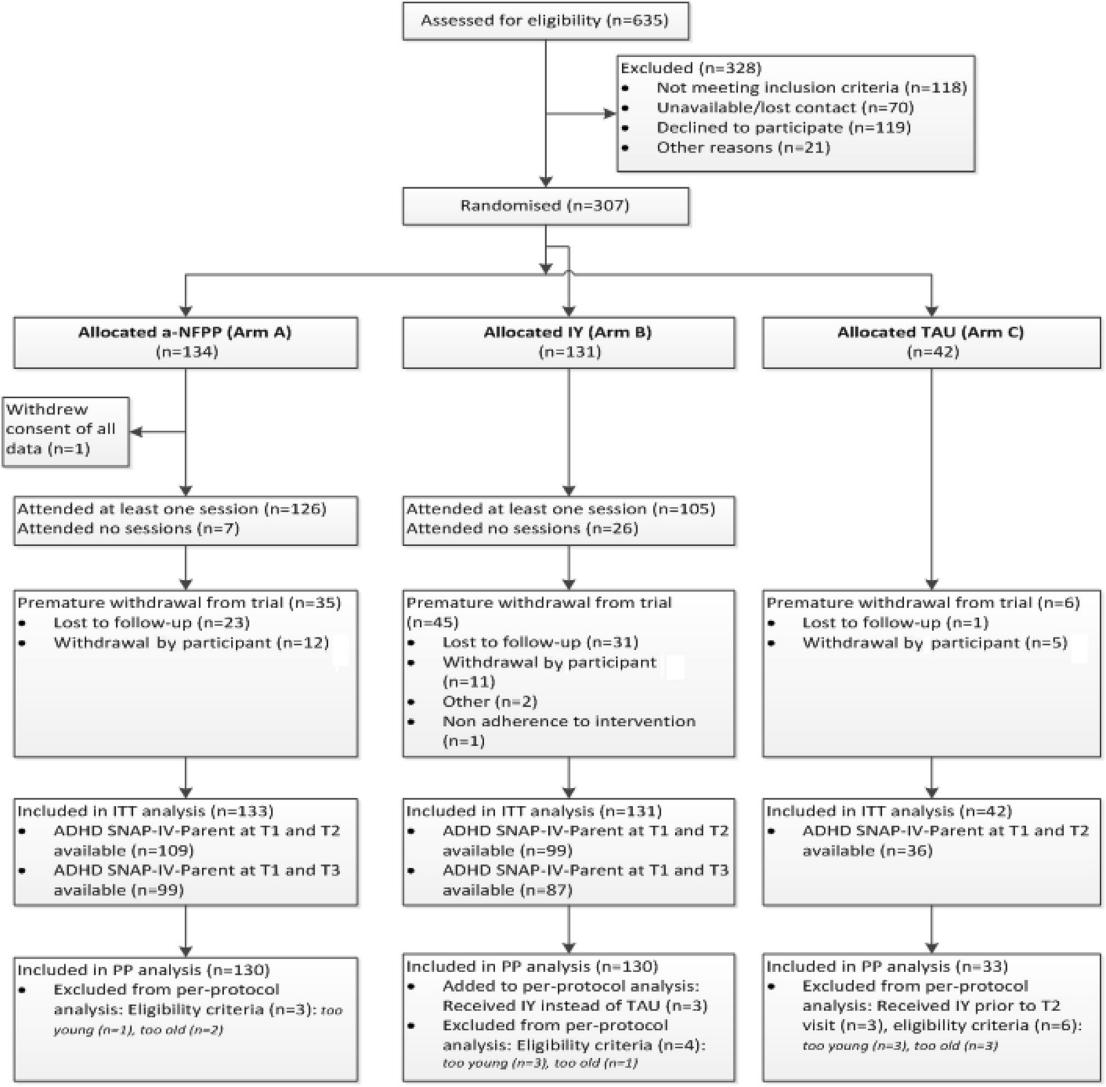
CONSORT diagram showing the flow of participants through the study

Three hundred and seven participants were recruited; one participant withdrew consent of all data (see Fig. 1). Per protocol analyses excluded 13 participants because of trial protocol violations and three because they received IY rather than TAU prior to T2 (Fig. 1).

IY had lower levels of initial engagement than NFPP; 19.8% (*n* = 26) of participants attended no sessions (i.e., had no intervention at all) compared to 5.3% (*n* = 7). Of those participants who attended the first session, 18% (*n* = 23) of IY participants withdrew prematurely compared to 22.2% (*n* = 26) for NFPP. More NFPP, than IY participants, attended eight or more sessions, 64.7% (*n* = 86) versus 46.6% (*n* = 61)—although for the IY intervention this was very unevenly distributed across the three centres (36–54%). Those remaining in the trial were not statistically different from those dropping out in terms of baseline characteristics (data available on request). Demographics and background characteristics of the sample at baseline were well balanced across arms (Table 1). Parents in the sample had relatively high levels of unemployment, single parenthood, educational underachievement and depressed mood. The number of parents who had GHQ scores of 11 or over (the accepted clinical threshold) were NFPP 100 (75.2%); IY 104 (79.4%); TAU 31 (73.8%). A substantial proportion of children displayed developmental delay [75% of the total sample, with 50% of the total sample displaying language delay (Table 1)].

**Table 1:** Demographic, background and baseline characteristics of participants in the three treatment arms (ITT population)

	NFPP (*n* = 133)^a^	IY (*n* = 131)^a^	TAU (*n* = 42)^a^
**Child characteristics**			
Age (months)—mean (SD)	43.4 (7.01)	42.0 (6.49)	42.3 (7.79)
Female—*n* (%)	32 (24)	38 (29)	12 (40)
Language delay—*n* (%)	56 (53)	54 (52)	21 (60)
Developmental delay—*n* (%)	92 (77)	88 (75)	25 (74)
Conduct problems—*n* (%)	104 (81)	101 (79)	26 (63)
**Parent/caregiver characteristics**			
Left school no qualifications—*n* (%)	23 (17)	13 (10)	4 (10)
Female—*n* (%)	129 (97)	126 (96)	40 (95)
Unemployed—*n* (%)	88 (66)	81 (62)	26 (62)
Partner unemployed—*n* (%)	19 (14)	19 (15)	8 (19)
Single-parent—*n* (%)	41 (31)	39 (30)	12 (29)
Low mood—*n* (%)	100 (75)	104 (79)	31 (74)
**Baseline measures**			
Child ADHD			
SNAP parent—mean (SD)	2.08 (0.51)	2.14 (0.48)	2.04 (0.45)
SNAP teacher—mean (SD)	1.16 (0.82)	1.33 (0.80)	1.17 (0.85)
Direct observation of attention—mean (SD)^a^	10.15 (4.76)	10.52 (4.18)	9.55 (3.22)
Child conduct problems			
SNAP parent—mean (SD)	2.03 (0.70)	2.01 (0.75)	1.97 (0.77)
SNAP teacher—mean (SD)	0.79 (0.85)	0.97 (0.83)	0.79 (0.74)
ECBI Intensity—mean (SD)	177.23 (30.80)	180.70 (35.57)	171.59 (32.25)
ECBI problem—mean (SD)^a^	22.10 (7.62)	22.53 (8.31)	19.49 (7.68)

Language delay and developmental delay were deemed present when an individual was at least 6 months behind their chronological age in relation to at least one milestone. Conduct problems were present with a score of 15 or more on the problem scale of parent rated Eyberg Child Behaviour Inventory; Low mood is defined as a score of 11 or more on the General Health Questionnaire (scores 0–3 for each item)

^a^All results obtained using models performed with multiple-imputed data

### Treatment as usual

TAU data were available for all but 6 (14.3%) participants. In the current trial, the content of TAU varied considerably. Many individuals received no treatment. Where they did receive some, it was typically of a non-specialised nature offered in child and adolescent clinics or in the community for families with a young child with ADHD. The use of health services during the trial was common but in most cases these were for general medical concerns and not for behavioural problems. Nine children visited child mental health services. Of these two children attended a special nursery and one a speech therapist. Two parents attended a general support group (Sure Start). In addition, six parents attending CAMHS received parent training for their children’s behaviour problem. In five cases, this was Triple-P which was offered at one of the sites and in one site, it was non-specific behavioural advice. One child had an assessment by an educational psychologist. Parents of six children had respite support by family members. No children in the study received medication for ADHD.

### Treatment fidelity

For NFPP, a random selection of 11% of cases was assessed for fidelity with each site contributing five individual whole sets of parent treatment sessions. For IY, data on content delivered were submitted for 53% of groups from two of the sites. Content fidelity was 70% for NFPP and 74% for the two IY sites for which results were available.

### Efficacy

#### NFPP versus IY

Table 2 reports mean scores for the primary and secondary outcomes by treatment arm at T2 and T3 for NFPP and IY for ITT analyses and the adjusted mean difference in outcome from the mixed effects regression models. At T2 (*n* = 24) 18.0% and (*n* = 32) 24.4% of primary outcome data were missing for NFPP and IY, respectively. No statistically significant differences between NFPP and IY were observed on parent assessed ADHD symptoms at T2 [adjusted mean for NFPP = 1.715, for IY = 1.724 mean difference − 0.009 95% CI − 0.191 to 0.173, *p* value 0.921; effect size (SMD) = 0.06]. For all secondary outcomes, differences between NFPP and IY were small and not statistically significant (all *p* values > 0.9; Table 2). Sensitivity analyses of the; (i) complete case set, (ii) per protocol sample and (iii) parents receiving eight or more sessions gave similar results (available on request).

**Table 2:** Primary and secondary outcomes immediately post treatment and at six months (ITT population)

Outcomes	Mean (standard deviation)^a^			NFPP–IY^b^		NFPP–TAU^b^		IY–TAU^b^	
	NFPP (*n* = 133)	IY (*n* = 131)	TAU^c^ (*n* = 42)	Adjusted mean difference (95% CI)	*p*	Adjusted mean difference (95% CI)	*p*	Adjusted mean difference (95% CI)	*p*
**Post treatment (T2)**									
ADHD									
SNAP parent^d^	1.70 (0.67)	1.76 (0.66)	1.83 (0.56)	−0.01 (−0.19 to 0.17)	0.92	−0.19 (−0.38 to 0.003)	0.053	−0.16 (−0.37 to 0.04)	0.121
SNAP teacher	1.13 (0.80)	1.20 (0.75)	1.19 (0.79)	0.001 (−0.21 to 0.21)	0.99	−0.05 (−0.47 to 0.37)	0.81	−0.05 (−0.40 to 0.30)	0.782
DOA	9.33 (3.23)	10.27 (4.39)	10.21 (3.40)	−0.69 (−1.80 to 0.43)	0.22	−1.08 (−2.25 to 0.10)	0.073	−0.23 (−1.85 to 1.40)	0.785
Conduct problems									
SNAP parent	1.55 (0.81)	1.66 (0.86)	1.74 (0.83)	−0.16 (−0.35 to 0.04)	0.11	−0.24 (−0.48 to −0.002)	0.048	−0.06 (−0.32 to 0.20)	0.658
SNAP teacher	0.73 (0.79)	0.83 (0.82)	0.67 (0.69)	−0.04 (−0.37 to 0.30)	0.83	0.14 (−0.31 to 0.59)	0.524	0.14 (−0.24 to 0.51)	0.467
ECBI Intensity	152.40 (40.71)	160.94 (43.56)	161.58 (34.83)	−4.62 (−14.58 to 5.33)	0.36	−13.05 (−25.90 to −0.19)	0.047	−9.08 (−20.94 to 2.78)	0.133
ECBI problem	16.16 (9.97)	17.22 (10.79)	18.83 (8.02)	−0.458 (−3.61 to 2.69)	0.77	−3.52 (−6.48 to −0.57)	0.019	−3.19 (−6.52 to 0.14)	0.061
**6 months (T3)**									
ADHD									
SNAP parent	1.76 (0.67)	1.73 (0.68)	–	0.04 (−0.14 to 0.23)	0.64	–	–	–	–
SNAP teacher	1.01 (0.74)	1.04 (0.70)	–	−0.05 (−0.33 to 0.24)	0.75	–	–	–	–
DOA	8.82 (4.00)	8.15 (2.96)	–	0.55 (−0.35 to 1.45)	0.23	–	–	–	–
Conduct problems									
SNAP parent	1.68 (0.86)	1.69 (0.88)	–	−0.04 (−0.25 to 0.18)	0.75	–	–	–	–
SNAP teacher	0.55 (0.68)	0.71 (0.70)	–	−0.15 (−0.45 to 0.15)	0.33	–	–	–	–
ECBI Intensity	159.53 (46.43)	159.56 (43.15)	–	3.77 (−6.06 to 13.60)	0.45	–	–	–	–
ECBI problem	17.00 (11.68)	15.98 (10.54)	–	2.10 (−0.73 to 4.94)	0.14	–	–	–	–

^a^From participants with complete data

^b^Results from mixed model on multiple-imputed data adjusted for baseline (T1), tranche and centre as fixed effects and therapist and participant as random effects

^c^TAU participants were followed up to T2 post treatment only and not to T3 6 months

^d^Primary endpoint

#### NFPP versus TAU

TAU T2 primary outcome data were missing for *n* = 6 (14.3%) participants of the primary outcome. Small benefits of NFPP over TAU were seen for parent-rated ADHD SNAP-IV scores (mean difference − 0.189 95%; CI − 0.380 to 0.003; SMD = 0.35)—effects just short of conventional levels of significance (*p* = 0.053). The effects on DOA also approached significance (*p* = 0.073; SMD = 0.37; Table 2). NFPP produced statistically significant reductions in conduct problems on all three parent-rated measures compared to TAU (all *p* values < 0.05; SNAP ODD − SMD = 0.34; ECBI intensity − SMD = 0.45; ECBI problem − SMD = 0.69; Table 2). No differences were seen for teacher-rated outcomes (all *p* values > 0.5). One NFPP-related adverse event was reported—an accidental minor head injury.

#### IY versus TAU

IY was not superior to TAU in terms of parent-rated ADHD (*p* = 0.121; Table 2), teacher-rated ADHD (*p* = 0.782; Table 2) or DOA (*p* = 0.785; Table 2). IY produced near statistically significant reductions in conduct problems for the ECBI measure compared to TAU (*p* = 0.061; Table 2). Complete case and per protocol analysis gave similar results (available on request).

### Costs

Table 3 reports the cost breakdown for the interventions. As discussed above following review of the raw data, some adjustments were made that reduced the cost of transport to supervisions and of crèches, as the high cost of both were a consequence of trial-specific arrangements which would not translate to a real-world setting (details available on request). The costs of créches will depend on the number of children attending as one worker has to be employed for every two children attending. In both cases, these adjustments reduced the IY cost estimates. Despite this, overall mean total cost was significantly lower for NFPP than IY (£1591 versus £2103) a difference of £512 (95% CI £324 to £700). The difference was almost entirely related to intervention costs (£1081 in NFPP versus £1569 in IY). As expected, therapist travel costs were more expensive for NFPP, while facility costs (crèches, halls and refreshments and parent travel costs) were greater for IY. More surprising were the higher preparation/supervision costs for IY. This partly arose from the need for additional supervision due to therapist changes and in part was due to trial recruitment difficulties involving an extension in trial length and the need to run additional groups (16 as opposed to the initially planned 15) that resulted in very small groups.

**Table 3:** Breakdown direct and indirect costs by arm

	NFPP (£)	IY (£)	NFP (%)	IY (%)
**Direct treatment costs**				
**Non recurre**nt				
Course fees/training	11,000	12,798	7.6	6.2
**Recurrent**				
Materials	5296	16,719	3.7	8.1
Preparation	21,497	35,330	14.8	17.2
Supervision	14,039	33,287	9.7	16.2
Therapist travel	24,152	11,789	16.7	5.7
Admin	27,841	28,142	19.2	13.7
Parent travel costs	4619	10,925	3.2	5.3
Crèche/refreshments	30	28,951	0.0	14.1
Delivery	36,434	27,581	25.1	13.4
Treatment delivery total	144,907	205,521	100.0	100.0
Number of Families (ITT)	134	131		
Average costs per family	1081	1569		
**Indirect costs**				
Health services	45,311	50,544		
Family Borne	23,067	19,427		
Overall total costs	213,286	275,492		
Overall costs per family	1591	2103		

## Discussion

This was the first trial to compare the efficacy and cost-effectiveness of an individually delivered ADHD PT programme (NFPP) with a group-based package (IY) as recommended on grounds of lower cost by NICE. We had two principal findings. First, NFPP was no more or less efficacious for preschool ADHD than IY. The apparent lack of superiority of NFPP over IY went against our expectations, as NFPP was designed specifically for the treatment of preschool ADHD while the IY version used was not. Second, NFPP was less costly to deliver than IY—even after taking into account differences in the initial supervision costs. Like NICE, we predicted that many components of the costs of group and individual PT would be similar but that these costs would be spread more thinly in IY. In hindsight, some of the cost differences observed in favour of NFPP might have been expected especially given the high premium placed on training and supervision by IY, and particularly when running a trial with naive therapists. To what extent do these trial-based cost estimates represent the real world? A recent IY study found that similarly high costs during a trial phase fell dramatically when rolled out in everyday care [32] as once therapists are trained supervision costs are less and training manuals do not need to be provided, although materials for parents are still required. Furthermore, it is possible in the community setting, that créches and lunches, and patient travel may not be needed. However, these estimates of IY as routine care were based on a different trial. No such trial exists for NFPP and we were unable to estimate equivalent costs in COPPI. Our cost comparisons should, therefore, be treated with some caution.

Whilst NFPP did show some benefits relative to TAU, especially for conduct problems, the effects on ADHD were lower than seen in prior NFPP trials [6, 11, 12]. One possible reason for the reduced effect of NFPP may lie with the composition of the sample. As mentioned in the methods section, our recruitment strategies together with the inclusion and limited exclusion criteria were designed to maximise the inclusion of a broad range of ADHD individuals. As a consequence, many of the children in the study were experiencing co-occurring difficulties and developmental delay and/or lived in difficult family circumstances and these may have made them more difficult to treat. This would also apply to the effects of IY, which were also less than would have been expected, although possibly accounted for by the high no show rate of 20%. The NFPP results were, however, consistent with recent meta-analyses [33]. The SMD for parent-rated ADHD was 0.35 in the current trial, which is similar to the SMD of 0.37 found in a recent meta-analysis for PT for ADHD. As in previous trials of PT, the parent-rated benefits of PT relative to TAU did not generalise to teacher ratings [11]. Parent ratings may over-inflate treatment effects because of the bias likely to be associated with parents’ lack of blindness and their investment in the therapeutic process. Alternatively, ratings may reflect real changes in children’s behaviour in the home setting that do not translate into improvement in other settings. Both IY and NFPP place a strong emphasis on improving the parent–child relationship so that behavioural improvements related to this therapeutic element might be especially likely to affect home rather school based outcomes. The (near significant) positive effects seen for NFPP compared to TAU on the direct observation measure of attention are more consistent with this latter account.

COPPI had considerable strengths relative to previous trials as the first RCT comparing group and individual PT for ADHD and, by some considerable margin, the largest ADHD PT trial to be conducted so far. These included a comparison with TAU and the use of teacher ratings and objective measures of change to examine issues of outcome blinding and generalisation. However, there were limitations. First, treatment attrition, particularly for the IY arm, was greater than planned for in the power calculation. Various strategies were employed to motivate families to remain in the trial including reimbursement of costs to parents for their time in completing questionnaires. This took the form of £5 gift vouchers for each set of complete data collected at baseline, T1 and T2. This was introduced roughly half way through the trial. Gift vouchers were handled by the researchers that collected the outcome data. However, it is possible that the challenging nature of the sample, as discussed above, could have contributed to the higher than expected levels of drop out. Importantly there was no evidence for selective drop out and so we were able to utilise a mixed effect regression model and multiple imputation to include information from all participants. Second, some elements of the IY implementation in COPPI may not have been optimised. Groups varied in terms of numbers of participants. IY developers recommend 12 individuals per group but most trials have started with eight—although numbers can drop during the treatment. The number of IY groups with less than 8 members at the time of recruitment was 13 out of 19—with a range 5–12 of participants per group and a modal value of 6/7. Furthermore, the twenty per cent of families that did not attend any sessions received no treatment. This high rate of IY “no shows” could be explained by: (i) parents failing to make the time/date of sessions (despite confirming their availability prior to randomisation); (ii) parents’ initial preference for individual over group approaches and/or (iii) difficulties in planning and organising their lives. Third, while overall IY content fidelity was acceptable for two sites (over 70%), data were not collected from one site. We do not know the full reason for this but it seems like it was due to a simple oversight. Staff changes made checking the return of ratings difficult. However, all therapists had attended supervision and their tapes had been discussed in supervision. Fourth, response rate for teachers was lower than that for parents. Fifth, there was a degree of turnover of staff in both programmes necessitating extra training and leading to increased costs. This was compounded by the late start of the trial in two centres and the need to run four additional tranches due to low recruitment [21]. Finally, it was not possible to estimate the costs of TAU. This meant that we were unable to make cost comparisons of this against IY and NFPP.

COPPI was designed to address the appropriateness of NICE guidance recommending group-based PT for ADHD in young children. The finding that NFPP may be less costly than IY supports a revision of NICE’s recommendations in favour of group rather than individual services. Arguably, both individually delivered and group-based PT should be made available to families of children with preschool ADHD [34]. An option of individually delivered PT is further supported by the higher rates of attrition for IY which may suggest that individual, rather than group-based approaches are preferred by some parents—a finding consistent with some, but not all, prior research and perhaps especially pertinent when working with potentially difficult to treat families where a high degree of structured flexibility in delivery is required [24]. Future efforts should focus on understanding parent preferences for different delivery approaches [34].

## Research in context

### Research before this study

We examined recent systematic reviews and meta-analyses of parenting training for ADHD (last published in 2014) and conducted a search using ISI Web of Knowledge and MEDLINE in August 2016 to identify recent RCTs. Search terms included—“parent training”, “Attention-Deficit Hyperactivity Disorder”, “ADHD”, “preschool”. Meta- analyses highlight the value of PT for pre-schoolers with ADHD—specifically improving parenting and reducing parent reported ADHD and conduct problem symptoms. No prior RCT has included a cost-effectiveness analysis of PT specifically for use in preschool ADHD populations. No prior study has compared the efficacy and cost-effectiveness of individually delivered and group-based parent training approaches.

### Added value of this study

This is the largest RCT of parent training as a treatment for preschool children with ADHD, the first to incorporate a cost analysis and the first to compare an individually delivered (NFPP) and a group-based (IY) approach. NFPP and IY did not differ from one another in terms of their effects on ADHD or conduct problems although, against expectation, in the trial context NFPP was less costly. Furthermore, attendance was higher in NFPP families.

### Limitations

The rate of attrition was higher than expected. The ability to check for fidelity of the delivery of IY in one of the centres was not possible. Aspects of IY delivery may not have been optimised. The cost estimates in the context of the trial may not reflect costs in routine care.

### Implications of all the available evidence

PT plays an important role in the treatment of preschool ADHD. Given it established that NFPP was equally effective to IY but likely to be cheaper, the current trial did not support the NICE recommendation in favour of group-based over individually delivered parent training for the treatment of preschool ADHD.

## References

[1] Faraone SV, Asherson P, Banaschewski T, Biederman J, Buitelaar JK, Ramos-Quiroga JA, Rohde LA, Sonuga-Barke EJS, Tannock R, Franke B (2015). Attention-deficit/hyperactivity disorder.

[2] Chorozoglou M, Smith E, Koerting J (2015). Preschool hyperactivity is associated with long-term economic burden: evidence from a longitudinal health economic analysis of costs incurred across childhood, adolescence and young adulthood. J Child Psychol Psychiatry.

[3] Chronis AM, Jones HA, Raggi VL (2015). Evidence-based psychosocial treatments for children and adolescents with attention-deficit/hyperactivity disorder. Clin Psychol Rev.

[4] Scott S, Dadds MR (2009). Practitioner review: when parent training doesn’t work: theory-driven clinical strategies. J Child Psychol Psychiatry.

[5] National Collaborating Centre for Mental Health (UK) (2009). Attention-Deficit/Hyperactivity Disorder: Diagnosis and Management of ADHD in Children.

[6] Sonuga-Barke EJ, Daley D, Thompson M (2001). Parent-based therapies for preschool attention-deficit/hyperactivity disorder: a randomized, controlled trial with a community sample. J Am Acad Child Adolesc Psychiatry.

[7] Jones K, Daley D, Hutchings J (2007). Efficacy of the Incredible Years basic parent training programme as an early intervention for children with conduct problems and ADHD. Child Care Health Dev.

[8] Niec LN, Hemme JL, Yopp JM, Brestan EV (2016). Group parent-child interaction therapy: a randomized control trial for the treatment of conduct problems in young children. J Consult Clin Psychol.

[9] Webster-Stratton C (1998). Preventing conduct problems in Head Start children: strengthening parenting competencies. J Consult Clin Psychol.

[10] Sonuga-Barke EJ, Thompson M, Abikoff H (2006). Nonpharmacological interventions for preschoolers with ADHD: the case for specialized parent training. Infants & Young Children..

[11] Abikoff HB, Thompson M, Laver-Bradbury C (2015). Parent training for preschool ADHD: a randomized controlled trial of specialized and generic programs. J Child Psychol Psychiatry.

[12] Thompson MJ, Laver-Bradbury C, Ayres M (2009). A small-scale randomized controlled trial of the revised new forest parenting programme for preschoolers with attention deficit hyperactivity disorder. Eur Child Adolesc Psychiatry.

[13] Webster-Stratton C (2008). The Incredible Years parents and toddlers series.

[14] Scott S, Spender Q, Doolan M (2001). Multicentre controlled trial of parenting groups for childhood antisocial behaviour in clinical practice. Br Med J.

[15] Hutchings J, Bywater T, Daley D, Gardner F, Whitaker C, Jones K, Eames C, Edwards RT (2007). Parenting intervention in Sure Start services for children at risk of developing conduct disorder: pragmatic randomised controlled trial. BMJ.

[16] Webster-Stratton CH, Reid MJ, Beauchaine T (2011). Combining parent and child training for young children with ADHD. J Clin Child Adolesc Psychol.

[17] Perrin EC, Sheldrick RC, McMenamy JM (2014). Improving parenting skills for families of young children in pediatric settings: a randomized clinical trial. JAMA Pediatr.

[18] Routh D, Magrab P (1978). Hyperactivity. Psychological management of pediatric problems.

[19] Costello EJ, Edelbrock CS, Costello AJ (1985). Validity of the NIMH diagnostic interview schedule for children: a comparison between psychiatric and pediatric referrals. J Abnorm Child Psychol.

[20] Jeffree DM, McConkey R (1998). Parent involvement project (PIP) developmental charts.

[21] McCann DC, Thompson M, Daley D (2014). Study protocol for a randomized controlled trial comparing the efficacy of a specialist and a generic parenting programme for the treatment of preschool. ADHD Trials.

[22] Koerting J, Smith E, Knowles MM (2013). Barriers to, and facilitators of, parenting programmes for childhood behaviour problems: a qualitative synthesis of studies of parents and professionals’ perceptions. Eur Child Adolesc Psychiatry.

[23] Smith E, Koerting J, Latter S (2015). Overcoming barriers to effective early parenting interventions for attention-deficit hyperactivity disorder (ADHD): parent and practitioner views. Child Care Health Dev.

[24] McEwan F, Thompson M, Laver-Bradbury C (2015). Innovations in practice: adapting a specialized ADHD parenting programme for use with ‘hard to reach’ and ‘difficult to treat’ preschool children. Child Adolesc Mental Health.

[25] Thompson MJJ (2002) The development of a community service for young children in the New Forest: joint work by a child guidance clinic with health visitors MD thesis. University of Glasgow (unpublished)

[26] Swanson J, Nolan W, Pelham WE (1992) The SNAP-IV rating scale. http://www.adhd.net. Accessed 25 Jul 2006

[27] Robinson EA, Eyberg SM, Ross AW (1980). The standardization of an inventory of child conduct problem behaviors. J Clin Child Adolesc Psychol.

[28] Beecham J, Knapp M, Thornicroft G (2001). psychiatric interventions. Measuring mental health needs.

[29] Goldberg D (1992). General health questionnaire (GHQ-12).

[30] Yang X, Shoptaw S (2005). Assessing missing data assumptions in longitudinal studies: an example using a smoking cessation trial. Drug Alcohol Depend.

[31] Dolan P (1997). Modeling valuations for EuroQol health states. Med Care.

[32] Edwards RT, Linck P, Berry R, Charles J, Jones C, Bywater T, Hutchings J (2016). Incredible Years parenting programme: cost-effectiveness and implementation. J Child Serv.

[33] Daley D, Van der Oord S, Ferrin M (2014). Behavioral interventions in attention-deficit/hyperactivity disorder: a meta-analysis of randomized controlled trials across multiple outcome domains. J Am Acad Child Adolesc Psychiatry.

[34] Wymbs FA, Cunningham CE, Chen Y, Rimas HM, Deal K, Waschbusch DA, Pelham WE (2015). Examining parents’ preferences for group and individual parent training for children with ADHD symptoms. J Clin Child Adolesc Psychol.

